# Synergistic effects of *Bifidobacterium animalis subsp. lactis* Ca360 and zinc sulfate on zinc transport, oxidative stress, and intestinal inflammation in zinc-deficient mice

**DOI:** 10.3389/fnut.2026.1786466

**Published:** 2026-05-28

**Authors:** Haofei Wang, Jing Yang, Linjun Wu, Xiangyu Bian, Xiaoqiong Li, Jian Kuang, Jianqiang Li, Fangshu Shi, Yuejian Mao, Jinjun Li, Haibiao Sun

**Affiliations:** 1 The First Hospital of Shanxi Medical University, Taiyuan, China; 2 State Key Laboratory for Quality and Safety of Agro-Products, Institute of Food Sciences, Zhejiang Academy of Agricultural Sciences, Hangzhou, China; 3 Global R&D Innovation Center, Inner Mongolia Mengniu Dairy (Group) Co. Ltd., Hohhot, China

**Keywords:** *B. lactis* Ca360, gut microbiota, probiotics, zinc absorption, zinc deficiency, zinc deficient mice

## Abstract

Zinc deficiency is closely associated with oxidative stress, inflammation, and intestinal dysfunction. In this study, a zinc-deficient mouse model was used to evaluate the effects of *Bifidobacterium animalis subsp. lactis* Ca360 (*B. lactis* 360) combined with ZnSO4 on zinc metabolism, antioxidant capacity, intestinal integrity, and gut microbiota composition. Zinc deficiency significantly reduced organ indices and zinc levels, impaired antioxidant enzyme activities, and induced oxidative stress and inflammation. Combined supplementation with *B. lactis* Ca360 and ZnSO4 markedly restored zinc status, enhanced superoxide dismutase and glutathione peroxidase activities, reduced nitric oxide levels, and alleviated colonic mucosal damage, with superior effects in several parameters compared with *Lactiplantibacillus plantarum* 299v (*L. plantarum* 299v). At the molecular level, *B. lactis* Ca360 upregulated duodenal zinc uptake and storage–related genes while suppressing pro-inflammatory gene expression. Moreover, *B. lactis* Ca360 reversed zinc deficiency–associated gut dysbiosis by reducing inflammation-related taxa and enriching Muribaculum, which was positively correlated with zinc status and antioxidant capacity. Overall, these findings demonstrate that *B. lactis* Ca360 combined with ZnSO4 effectively mitigates zinc deficiency–induced oxidative stress and intestinal dysfunction, highlighting its potential as a targeted probiotic strategy for managing zinc deficiency–related disorders.

## 1 Introduction

Zinc, as an essential trace element for living organisms, participates in biochemical reactions of over 300 enzymes and transcription factors, and is crucial for cell division, immune regulation, and protection against oxidative damage (1, 2). Zinc plays an important role in multiple components of the immune system, and its anti-inflammatory activity, antibacterial properties, as well as participation in organ function recovery are all very important (3). The global issue of zinc deficiency is prominent, which can lead to immune dysfunction, increased oxidative stress, and increased risk of neurodegenerative diseases, chronic kidney disease, and other conditions (4, 5). Although traditional zinc supplements such as zinc sulfate is widely used, they have limitations such as low absorption rates and gastrointestinal discomfort (6). Therefore, novel and safer treatment methods are needed.

In recent years, research has demonstrated that the gut microbiota significantly influences human health, with its dysbiosis being linked to a spectrum of disorders, including inflammatory bowel diseases, obesity, diabetes, and cardiovascular diseases (7). The gut microbiota, by modulating host immune responses, metabolic pathways, and inflammatory states, presents a potential therapeutic target for conditions like zinc deficiency (8). Zinc metabolism is closely related to the gut microbiota, and previous studies have shown that zinc homeostasis is partially dependent on the gut microbiota (9, 10). The gut microbiota may modulate zinc metabolism through its impact on key physiological processes, including immune and inflammatory responses, as well as zinc-related signaling pathways along the gut-zinc axis (11). Furthermore, the gut microbiota generates key metabolites, such as short-chain fatty acids (SCFAs), that are essential for preserving intestinal barrier function, thereby significantly influencing overall human health (12). This evidence suggests that interventions in gut microbiota and related metabolite levels can help improve the outcomes of zinc deficiency.

*Bifidobacterium lactis* may have anti-inflammatory, antioxidant, and immunomodulatory properties, which may contribute to disease prevention and treatment (13, 14). In fact, *Bifidobacterium lactis* have shown beneficial effects by regulating the balance of gut microbiota (15). Previous studies have demonstrated that *Bifidobacterium lactis* exhibits a satisfactory safety profile and is generally well-tolerated, with minimal side effects (16–18). *Bifidobacterium lactis* Ca360 (*B. lactis* Ca360) not only has a strong ability to increase short-chain fatty acids and high phytase activity, but more importantly, at the functional level, it can simultaneously and efficiently promote the absorption of three key minerals—calcium, iron, and zinc—in intestinal models and has good safety (19). However, the effects of *B. lactis* Ca360 on the adjustment of gut microbiota composition, the production of metabolites by gut microbiota, the effects on zinc metabolism in the body, and the effects on organ inflammation damage caused by zinc deficiency are still unknown.

This study aims to explore the effect of *B. lactis* Ca360 and ZnSO4 complexes ( on zinc deficiency using a combination of LEfSe and Spearman correlation analysis, 16S rDNA gene sequencing, and qPCR technology. According to previous research, the use of *L. plantarum*299v fermentation represents an effective strategy to notably improve the accessibility of minerals in pseudocereal flours (20). Furthermore, a study reported that *L. plantarum* 299v is effective in enhancing mineral iron absorption, so it was chosen as a control for *B. lactis* Ca360 (21). This study utilized various types of zinc supplements, including ZnSO4, *L. plantarum* 299v and ZnSO4 complexes, as well as *B. lactis* Ca360 and ZnSO4 complexes, and proposed the mechanism by which *B. lactis* Ca360 affects zinc metabolism, which may include regulation of inflammatory cytokine levels, regulation of gut microbiota, and changes in gene expression. This study may enhance our understanding of the relationship between gut microbiota and zinc metabolism, and propose new approaches for treating zinc deficiency.

## 2 Materials and methods

### 2.1 Animal models and therapeutic interventions

A total of 50 male Kunming mice (KM mice), with an average body weight of 11.0 ± 0.2 g, were obtained from GemPharmatech Co., Ltd. (Jiangsu, China). All mice were maintained under standard laboratory conditions with a stable ambient temperature of 23–25 °C and a 12-h light/dark cycle. After 1 week of adaptive feeding, the mice were randomly divided into five groups: normal control group(fed with a normal mouse diet), model group (fed with a Zn-deficient diet) and three treatment groups [fed with a Zn-deficient diet supplemented daily by gavage with ZnSO4 (6.96 mg Zn/kg body weight) (22), *L. plantarum* 299v (1x10^9^ cfu/ml) supplemented with ZnSO4 complexes solution (6.96 mg Zn/kg body weight), and *B. lactis* Ca360 (1x10^9^ cfu/ml) supplemented with ZnSO4 complexes solution (6.96 mg Zn/kg body weight)] (23). *B. lactis* Ca360 is an excellent strain obtained from previous *in vitro* screening studies, demonstrating no cytotoxicity, strong phytase activity (which may aid in mineral liberation), and the ability to increase the production of short-chain fatty acids (SCFAs)—properties that may confer advantages in mineral absorption and gut health modulation compared to some other strains. According to unpublished experiments, *B. lactis* Ca360 has no cytotoxicity, strong phytase activity, and can increase the production of SCFA. *L. plantarum* 299v and *B. lactis* Ca360 is provided by Mengniu Co., Ltd (Neimenggu, China). The experiment lasted for a period of 3 weeks. Throughout the study, all mice were provided with deionized water *ad libitum* and monitored daily for general health conditions (24). At the end of the experiment, fecal samples were collected and mice in each group were subjected to a 12-h fast and then anesthetized via an intraperitoneal injection of pentobarbital sodium at a dosage of 50 mg/kg body weight. The anesthetized mice were placed in a supine position and secured on a wax board. An abdominal incision was made to immediately expose the abdominal cavity. The heart, liver, spleen, and kindey were removed and washed repeatedly with normal saline. After the excess water was dried with absorbent paper, the organ wet weight was obtained. All animal procedures were performed in accordance with the guidelines and regulations approved by the Animal Ethics Committee of Zhejiang Academy of Agricultural Sciences (Approval Number: 25ZALAS11). The formula for calculating the organ index is as follows:


$$\begin{array}{l} Organ\;index\;\left(\%\right)=\frac{Organ\;weight\;\left(g\right)}{Body\;weight\;\left(g\right)}\times 100\% \end{array}$$ (1)

### 2.2 Biochemical analysis and cytokine measurements

Serum was separated from blood samples via centrifugation at 2,000 × g for 10 min at 4 °C and stored at −80 °C for subsequent analysis. The concentrations of zinc and nitric oxide (NO), as well as the activities of glutathione peroxidase (GPx) and superoxide dismutase (SOD) in the serum, were assayed using commercial kits (Zinc/NO/SOD/GPx assay kits, Catalog Number AKBL007M/AKNM005M/AKAO001M-100S/AKPR014M, Boxbio Science & Technology Co., Ltd., Beijing, China). Meanwhile, liver tissues were homogenized (1:9, w/v) in ice-cold normal saline (0.9%) and centrifuged at 6,000 × g for 15 min at 4 °C. The resulting supernatant was collected to determine zinc content and NO levels, along with GPx and SOD activities.

### 2.3 Colon histology analysis

Colon tissue sections were fixed overnight in 4% paraformaldehyde (PFA) to preserve tissue architecture and antigenicity. Subsequently, the fixed sections were processed for routine Hematoxylin and Eosin (H&E) staining to evaluate histopathological changes. Colon histology was assessed in a blinded manner on sections from all mice in each group, and the presented images are representative of the consensus morphological changes observed. The detailed staining protocol has been described previously (25).

### 2.4 Fecal 16S rRNA gene sequencing analysis and short-chain fatty acid analysis

Fecal samples were homogenized in phosphate-buffered saline (PBS) at a 1:10 (w/v) ratio and thoroughly vortexed. After centrifugation, the supernatant was collected, filtered, and analyzed for short-chain fatty acids (SCFAs) using a GC-2010 Plus gas chromatograph (Shimadzu Corporation, Kyoto, Japan) equipped with a DB-FFAP column (Agilent Technologies, Santa Clara, CA, USA), following established protocols (25).

For microbial community profiling, total genomic DNA was extracted from cryopreserved (−80 °C) fecal samples. The V3–V4 hypervariable region of the bacterial 16S rRNA gene was amplified with universal primers 341F and 805R, and paired-end sequencing was performed on an Illumina platform (26). The 5′ ends of the primers were tagged with specific barcodes per sample and sequencing universal primers. PCR amplification was performed in a total volume of 25 μL reaction mixture containing 25 ng of template DNA, 12.5 μL PCR Premix, 2.5 μL of each primer, and PCR-grade water to adjust the volume. The PCR conditions to amplify the prokaryotic 16S fragments consisted of an initial denaturation at 98 °C for 30 s; 32 cycles of denaturation at 98 °C for 10 s, annealing at 54 °C for 30 s, and extension at 72 °C for 45 s; and then a final extension at 72 °C for 10 min. The PCR products were confirmed with 2% agarose gel electrophoresis. Throughout the DNA extraction process, ultrapure water, instead of a sample solution, was used to exclude the possibility of false-positive PCR results as a negative control. The PCR products were purified by AMPure XT beads (Beckman Coulter Genomics, Danvers, MA, USA) and quantified by Qubit (Invitrogen, USA). The amplicon pools were prepared for sequencing and the size and quantity of the amplicon library were assessed on Agilent 2100 Bioanalyzer (Agilent, USA) and with the Library Quantification Kit for Illumina (Kapa Biosciences, Woburn, MA, USA), respectively. The libraries were sequenced on NovaSeq PE250 platform. Raw sequencing data were processed using QIIME2 (version 2022.11). Quality filtering, denoising, and chimera removal were performed using the DADA2 plugin. Taxonomic classification was conducted against the SILVA database (version 138).

### 2.5 Quantitative real-time reverse transcription–PCR analysis

In this study, a systematic molecular biological method was used to analyze the gene expression of ileal tissue samples: first, about 20 mg of tissue was ground in liquid nitrogen, and then the total RNA was extracted by TransGen Biotech kit. After purification by chloroform isopropanol method, the RNA purity (A260/A280 ratio 1.8–2.0) was detected by spectrophotometer, and the integrity was verified by agarose gel electrophoresis; Then 1 μg of RNA was used to synthesize cDNA with primers and reverse transcriptase; qPCR reaction was carried out in 20 μL system by SYBR green fluorescent dye method, including 2 × SYBR premix, 10 μm forward and reverse primers and cDNA template. The reaction procedure included pre denaturation at 94 °C for 30 s, amplification at 94 °C for 5 s and amplification at 50–60 °C for 15 s with 40–45 cycles (fluorescence was collected at this stage), and then the specificity was verified by melting curve analysis; Take Gapdh mRNA as the internal reference gene, use the primers shown in Table S1, and repeat all experiments three times to ensure the reliability of the data (27–29). The relative mRNA expression levels of target genes were calculated using the 2^−ΔΔ^Ct method, normalized to the housekeeping gene Gapdh.

### 2.6 Statistical analysis

Data analyses were performed using GraphPad Prism 10.1.2 (GraphPad Software, USA). All data are presented as mean values ± standard error of the mean (SEM). Student's t-test was used for comparison between two groups, and one-way ANOVA was used for comparison among multiple groups. Significance levels are indicated as follows: ^*^p < 0.05, ^**^p < 0.01, ^***^p < 0.001.

## 3 Results

### 3.1 The combination of *B. lactis* Ca360 and ZnSO4 ameliorates organ indices and oxidative stress related zinc metabolism in zinc-deficient mice

**Figure 1:**
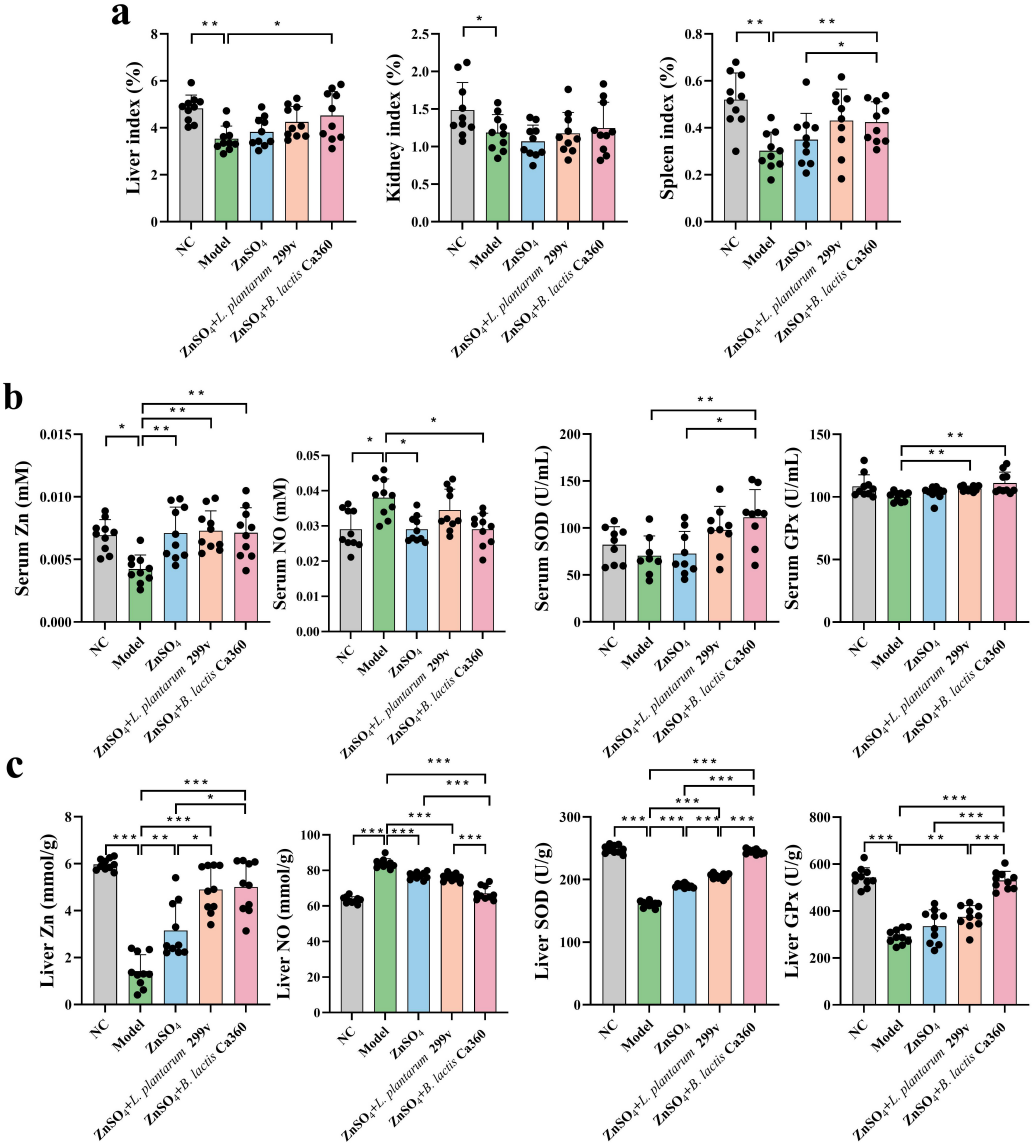
Effects of *B. lactis* Ca360 combined with ZnSO_4_ on **(a)** organ indices, **(b)** zinc status, and **(c)** oxidative stress in zinc-deficient mice. Data are presented as mean ± S.E.M. Significance levels are indicated as follows: *p < 0.05, **p < 0.01, ***p < 0.001.

Compared with the normal control (NC) group, mice in the Model group showed a significantly reduced liver, kidney, and spleen indices. Inventention with ZnSO4 patially restored these indices across all treatment groups. Notably, the combination of *B. lactis* Ca360 and ZnSO4 significantly higher liver and spleen indices compare to the Model group, bringing them close to NC levels. Moreover, the improvement in spleen index was significantly greater than that with ZnSO4 alone and was also superior to that of the *L. plantarum* 299v combined intervention group (Figure 1a). However, none of the intervention groups improved the kidney indices. As shown in Figure 1b, compared with the NC group, the Model group showed a significant decrease in serum Zn deficiency and elevated, indicating severe microelement imbalance and oxidative stress. Intervention with combination of *B. lactis* Ca360 and ZnSO_4_ effectively reversed these patholohical changes. Specifically, serum Zn concentration was significantly restored, SOD and GPx activities were markedly enhaced, NO levels were substantially reduced (Figure 1b). Notely, these metabolic recoveries were supior in magnitude to those observed in the *L. plantarum* 299v combined intervention group, underscorcing the superior metabolic regulatory advantage of *B. lactis* Ca360 in liver injury repair.

Figure 1c illustrates hepatic metabolic parameters. Compared with the NC group, mice in the Model group exhibited a significant reduction in hepatic Zn content, a marked decrease in SOD and GPx activities, and a substantial elevation in NO levels. The *B. lactis* Ca360 combined intervention demonstrated restorative effects. Compared to the Model group, the *B. lactis* Ca360 combined intervention significantly restored hepatic Zn content - an effect that also surpassed that achieved by ZnSO4 alone. Furthermore, the *B. lactis* Ca360 combined intervention significantly enhanced the activities of hepatic SOD and GPx compare to both the Model group and the *L. plantarum* 299v combined intervention group, while simultaneously and significantly suppressing NO levels relative to these two groups.

### 3.2 The combination of *B. lactis* Ca360 and ZnSO4 alleviates colon structural damage in zinc-deficient mice

**Figure 2:**
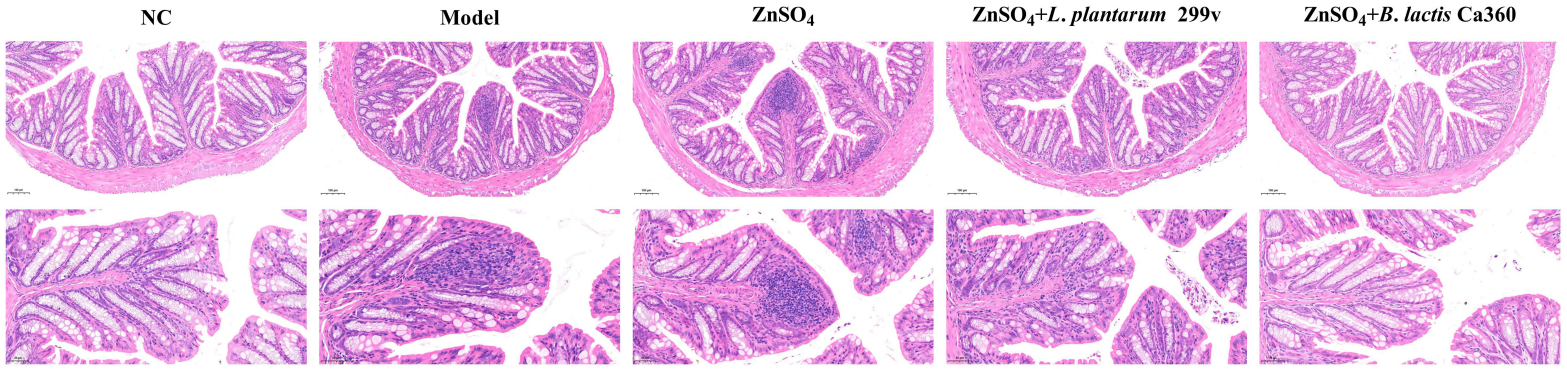
H&E staining of colon tissues from different groups at different magnifications. Representative H&E-stained colon sections from NC, Model, ZnSO_4_, ZnSO_4_+*L. plantarum* 299v, and ZnSO_4_+*B. lactis* Ca360 groups, scale bar 50 μm.

Figure 2 presents the hematoxylin and eosin (H&E) staining results of colonic tissues. The colonic architecture in the NC group appeared intact, featuring regularly arranged mucosal epithelial cells, well-defined glandular structures, preserved crypt morphology, and no evident inflammatory cell infiltration. In contrast, the Model group exhibited pronounced colonic tissue damage, characterized by disorganized glandular arrangement, partial disruption of crypt structures, and extensive inflammatory cell infiltration. Following ZnSO4 intervention, these colonic histopathological alterations were partially alleviated, though substantial inflammatory cell infiltration persisted. Notely, both the *L. plantarum* 299v and *B. lactis* Ca360 interventions led to further restoration of crypt architecture and a marked reduction in inflammatory infiltration. Importantly, the improvement observed in the *B. lactis* Ca360 combination group was particularly pronounced, with colonic tissue morphology closely resembling that of the NC group.

### 3.3 The combination of *B. lactis* Ca360 with ZnSO4 restores the expression of zinc homeostasis and inflammation-related genes in the duodenum of zinc-deficient mice

**Figure 3:**
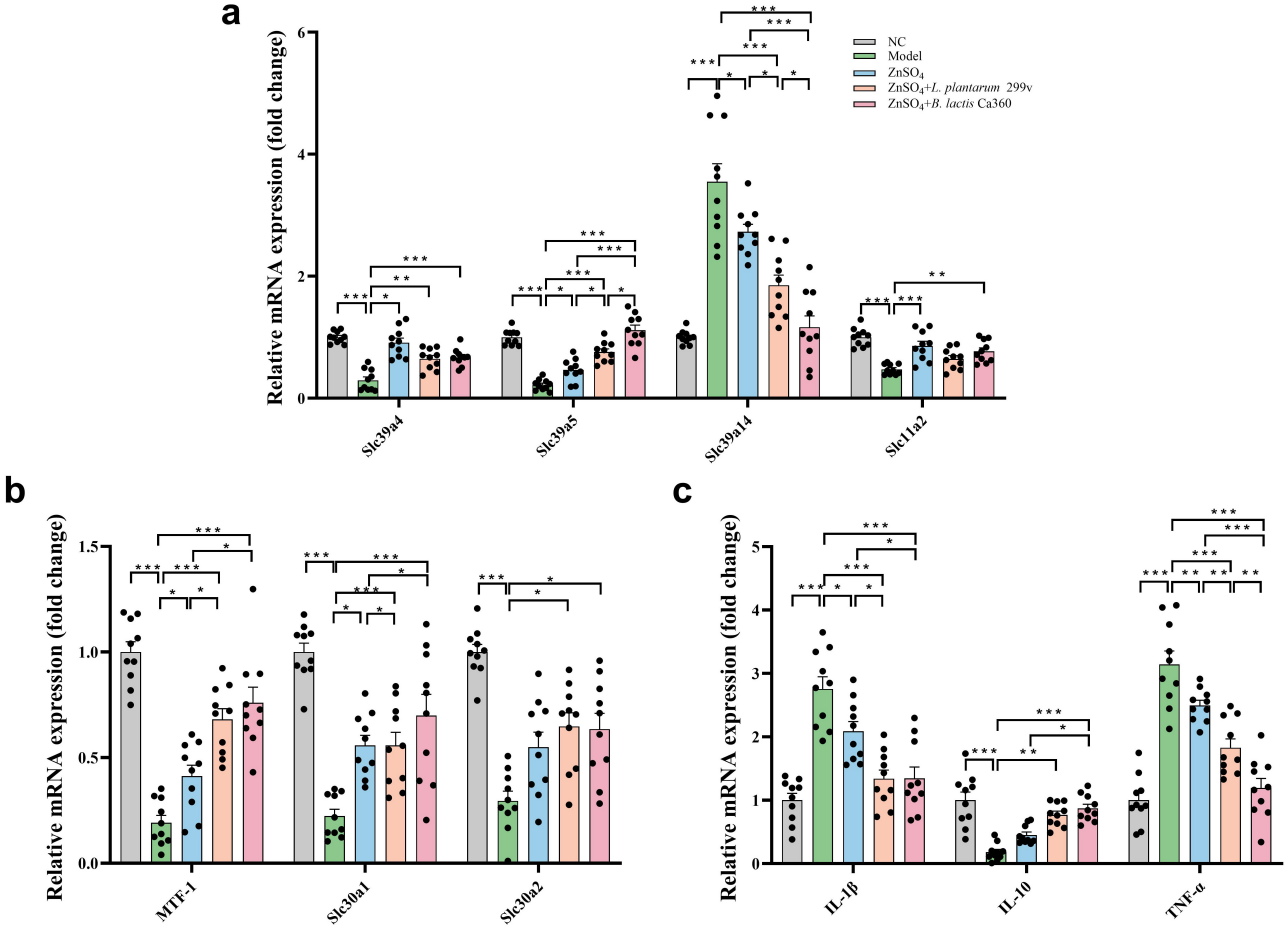
Effects of *B. lactis* Ca360 combined with ZnSO4 on duodenal gene expression in zinc-deficient mice **(a)** zinc absorption-related genes **(b)** zinc storage and homeostasis-related genes **(c)** inflammation-related genes. Each group consisted of *n* = 10 mice. Data are presented as ‘mean ± SEM'. Significance levels are indicated as follows: *p < 0.05, **p < 0.01, ***p < 0.001.

Compared with the NC group, the Model group exhibited significant dysregulation of duodenal genes related to zinc uptake, storage, and inflammation. Specifically, mRNA levels of *Slc39a4, Slc39a5, Slc11a2, MTF-1, Slc30a1*, and *Slc30a2* were significantly downregulated, whereas *Slc39a14, IL-1*β, and *TNF-*α were markedly upregulated. Expression of *IL-10* was also significantly reduced (Figures 3a–c). ZnSO4 intervention partially reversed these alterations across all treatment groups relative to the Model group. Notably, the combination of *B. lactis* Ca360 with ZnSO4 demonstrated superior efficacy in restoring the expression of key genes. It induced a significantly stronger restoration of *Slc39a5* and *Slc30a1* expression than the *L. plantarum* 299v combined intervention. Furthermore, the downregulation of *Slc39a14* and *TNF-*α was more pronounced in the *B. lactis* Ca360 combined intervention group, collectively resulting in a duodenal gene expression profile that more closely resembled that of the NC group.

### 3.4 *B. lactis* Ca360 combined with ZnSO4 reverses zinc deficiency-related microbial dysbiosis and enriches specific beneficial bacterial groups

**Figure 4:**
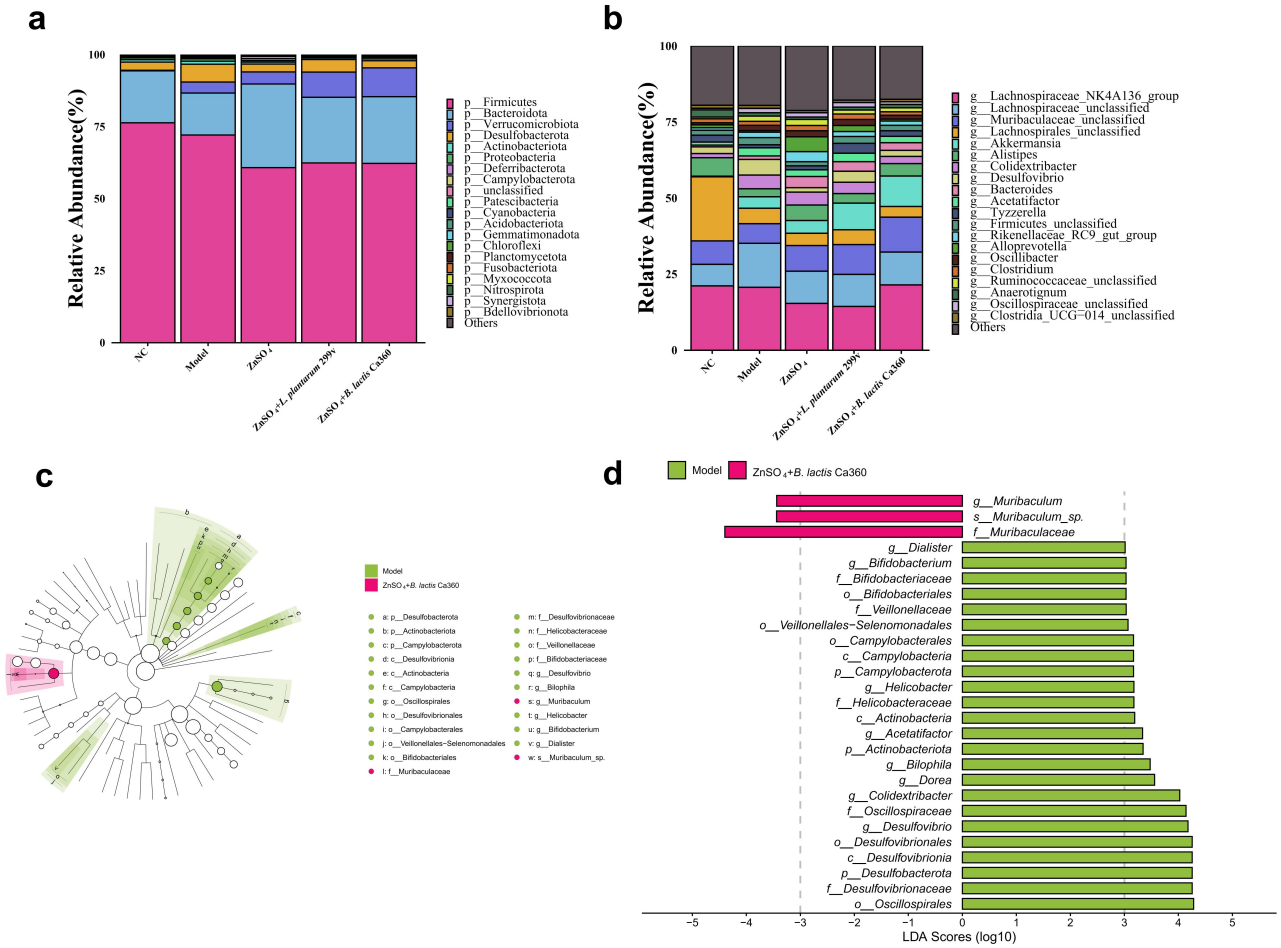
Gut microbiota composition and analysis results of 16S rDNA sequencing. **(a)** community structures of the observed samples at the phylum levels. **(b)** community structures of the observed samples at the genus levels. **(c, d)** LEfSe analysis, LDA ≥3.0. Each group consisted of *n* = 10 mice.

The compositional shifts of the gut microbiota are shown in Figure 4a. At the phylum level (Figure 4a), compared with the NC group, the Model group exhibited decreased relative abundances of *Firmicutes* and *Bacteroidota*, accompanied by a marked increase in *Desulfobacterota*. *B. lactis* Ca360 combined intervention significantly counteracted these changes by elevating the relative abundances of *Bacteroidota* and *Verrucomicrobiota*, while significantly reducing *Firmicutes* and *Desulfobacterota*. relative to the Model group. At the genus level (Figure 4b), further structural alterations were observed. Compared with the NC group, the Model group showed significantly decreased relative abundances of *Alistipes, Tyzzerella*, and *Anaerotignum*, while the relative abundances of *Colidextribacter, Desulfovibrio, Acetatifactor*, and *Akkermansia* were markedly elevated. Both *B. lactis* Ca360 and *L. plantarum* 299v interventions significantly ameliorated these genus-level alterations relative to the Model group; however, the overall regulatory effect was more pronounced in the *B. lactis* Ca360 + ZnSO4 group. Furthermore, LEfSe analysis distinct microbioal biomarkers associated with specific groups. *Muribaculum* was identified as the dominant biomarker genus in the *B. lactis* Ca360 group. In contrast, whereas in the Model group was characterized by a set of biomarker genera including *Colidextribacter, Desulfovibrio, Acetatifactor, Dorea, Helicobacter*, and *Dialister* (Figures 4c, d). In the Supplementary materials, α diversity analysis showed that there were no significant differences in the Chao1 index and Shannon index among the groups, indicating that the species richness and species diversity were similar across groups. The β diversity analysis results showed that all intervention groups significantly altered the microbial community structure of the mice.

### 3.5 *B. lactis* Ca360 combined with ZnSO4 regulates the production of SCFAs in zinc deficient mice

**Figure 5:**
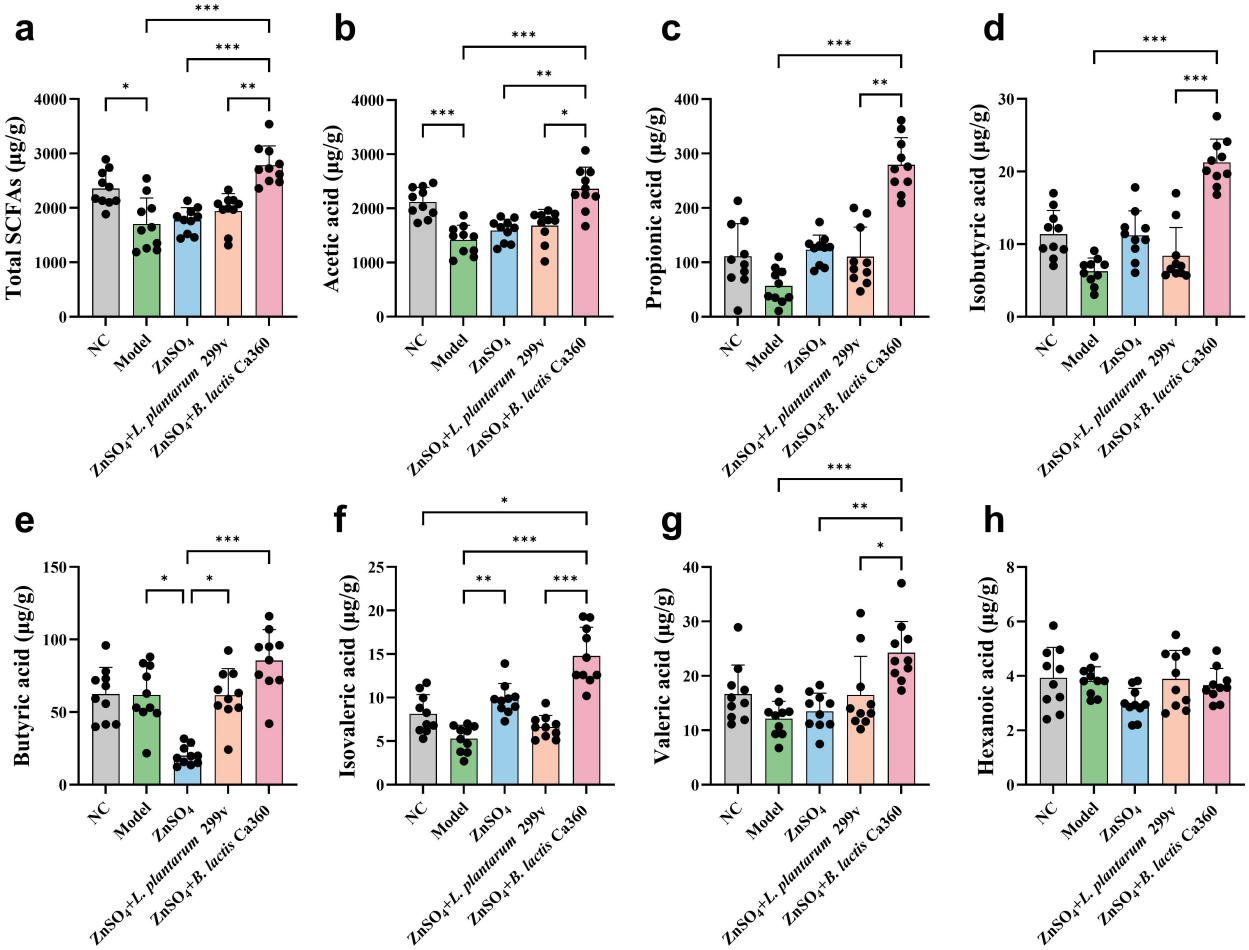
*B. lactis* Ca360 cominẉith ZnSO4 regulates the production of SCFAs in zinc deficient mice. **(a)** Total SCFAs, **(b)** acetate, **(c)** propionate, **(d)** isobutyrate, **(e)** butyrate, **(f)** isovalerate, **(g)** valerate, and **(h)** hexanoate. Each group consisted of *n* = 10 mice. Data are presented as ‘mean ± SEM'. Significance levels are indicated as follows: *p < 0.05, **p < 0.01, ***p < 0.001.

The gas chromatography analysis in Figure 5 shows significant changes in fecal short-chain fatty acid (SCFA) production. The total SCFA concentration (Figure 5a), acetate concentration (Figure 5b), propionate concentration (Figure 5c), isobutyrate concentration (Figure 5d), isovalerate concentration (Figure 5f), and valerate concentration (Figure 5g) in the *B. lactis* Ca360 + ZnSO4 group were significantly higher than those in the zinc-deficient group. Although the ZnSO4 group and the *L. plantarum* 299v + ZnSO4 group showed similar effects, their impact was significantly weaker than that of the *B. lactis* Ca360 + ZnSO4 group. Notably, the butyrate concentration in the ZnSO4 group (Figure 5e) was significantly lower than in the other groups, while the hexanoate concentration (Figure 5h) showed no significant differences among the groups. These results suggest that *B. lactis* Ca360 combined with ZnSO4 can significantly regulate SCFA production.

### 3.6 Correlation of gut microbiota with markers of zinc metabolism, oxidative stress, SCFAs and inflammation

As shown in Figure 5, Spearman correlation analysis was performed on organ indices, zinc metabolism markers, oxidative stress parameters, duodenal gene expression, SCFAs, and key differential bacterial genera. Notably, serum and hepatic Zn levels were significantly positively correlated with liver and spleen indices and the activities of antioxidant enzymes SOD and GPx, while negatively correlated with NO levels. This pattern was also observed in the expression of zinc transporters (Slc39a4, Slc39a5, Slc11a2, Slc30a1, and Slc30a2) and the transcription factor MTF-1, which were positively correlated with zinc content and antioxidant capacity but negatively correlated with NO. Additionally, the production of total SCFAs, acetate, propionate, butyrate, isobutyrate, valerate, and isovalerate was likewise positively correlated with zinc content and antioxidant capacity but negatively correlated with NO. In contrast, the expression of Slc39a14 and pro-inflammatory cytokines (IL-1β and TNF-α) was positively correlated with NO and negatively correlated with zinc content, SCFA production, and antioxidant enzyme activity, whereas the expression of IL-10 was significantly positively correlated with zinc and antioxidant markers.

At the microbial level, the relative abundances of *Muribaculum, Colidextribacter*, and *Dorea* were positively correlated with zinc status, antioxidant activity, SCFA production, and beneficial gene expression, while negatively correlated with NO and pro-inflammatory cytokines (IL-1β and TNF-α). Conversely, *Desulfovibrio, Bilophila, Helicobacter*, and *Dialister* showed the opposite correlation pattern, being positively correlated with inflammation and oxidative indicators, but negatively correlated with zinc, SCFA production, and antioxidant indices.

## 4 Discussions

Zinc is an essential trace element required for normal growth and development, immune function, and the maintenance of redox homeostasis (30, 31). Prolonged zinc deficiency is known to induce multi-organ dysfunction, disrupt oxidative balance, and impair intestinal barrier intergrity (32, 33). In this study, a zinc-deficient mouse model was established to systematically evaluate the effects of combined supplementation with *B. lactis* Ca360 and ZnSO4 on zinc metabolism, systemic oxidative stress, intestinal histopathology, duodenal gene expression, and gut microbiota composition.

At the systemic level, zinc-deficient mice exhibited significantly reduced organ indices (liver, kidney, and spleen), reflecting impaired organ development and function due to zinc deficiency, consistent with previous reports associating zinc deficiency with hepatic metabolic disorders and immune organs atrophy (34). Although ZnSO4 supplementation alone partially ameliorated these impairments, the combined intervention with *B. lactis* Ca360 resulted in more pronounced improvements in liver and spleen indices and demonstrated superior efficacy in certain parameters compared with combined intervention with *L. plantarum* 299v. Previous studies have demonstrated that specific probiotic strains can enhance zinc supplementation efficacy by improving mineral bio-availability or intestinal absorption efficiency, and the present findings further support the existence of strain-specific differences in synergistic zinc interventions (35). In terms of zinc metabolism and redox homeostasis, zinc-deficient mice showed significantly lowered serum and hepatic zinc levels, accompanied by elevated NO levels and decreased activities of antioxidant enzymes SOD and GPx, indicating a marked disruption of redox balance. These results are consistent with known roles of zinc in maintaing the structural and and function of key antioxidant enzymes (36). ZnSO4 partially restored serum zinc levels and reduced NO accumulation in zinc-deficient model animals, suggesting that traditional inorganic zinc supplementation retains a fundamental role in correcting zinc deficiency. However, its capacity to regulate the serum antioxidant enzyme system proved limited, failing to significantly enhance SOD and GPx activity. This indicates that zinc supplementation alone struggles to effectively reverse the systemic oxidative stress state associated with zinc deficiency. In contrast, combined intervention with *B. lactis* Ca360 not only demonstrated superior efficacy in restoring zinc levels but also markedly enhanced SOD and GPx activity in both serum and liver tissues while effectively suppressing excessive NO production. This suggests it may exert more comprehensive protective effects by improving zinc utilization efficiency within the body and synergistically regulating the antioxidant defense system.

Histological analysis further confirmed that zinc deficiency severely damaged colonic mucosal architecture, characterized by crypt morphology and increased inflammatory cell infiltration. These findings consistent with prior studies indicating that zinc deficiency compromises intestinal barrier integrity and promotes inflammation responses (37). Compared to the model group, zinc-deficient mice showed alleviated colonic damage and reduced inflammation after ZnSO4 intervention. Although ZnSO4 supplementation alone partially alleviated these sturctural abnormalities, the combined intervention with *B. lactis* Ca360 or *L. plantarum* 299v resulted in more pronounced restoration of crypt structure and greater reduction in inflammatory infiltration. This suggests that traditional inorganic zinc supplements ZnSO4 have very limited improvement effects on intestinal inflammation and damage caused by zinc deficiency, while specific probiotics may synergistically promote the repair and homeostasis of intestinal tissues in zinc deficiency. At the molecular level, zinc deficiency led to a marked supression of several key genes involved in zinc uptake and storage (*Slc39a4, Slc39a5, Slc11a2, MTF-1, Slc30a1*, and *Slc30a2*) in the duodenum, accompanied by upregulation of *Slc39a14* and the pro-inflammatory cytokines (*IL-1*β and *TNF-*α), and downregulation of the anti-inflammatory cytokine (*IL-10*). These transcriptional alterations align with estabilished evidence that members of the *Slc39* (ZIP) and *Slc30* (ZnT) families are critical for maintaining intestinal zinc absorption and intracellular zinc homeostasis, while inflammatory states can further disrupt these zinc transport pathways through cytokine-mediated signaling (38). ZnSO4 only has a significant effect on regulating the expression of *Slc39a4* and *Slc11a2* compared to the combination of *B. lactis* Ca360 and ZnSO4, while it has no significant effect on regulating the expression of *Slc39a5, Slc39a14, MTF-1, Slc30a1, IL-1*β, and *TNF-*α. Moreover, there was no significant difference in the role of ZnSO4 in regulating the expression of *Slc30a2* and *IL-10* compared to the model group. The effect of the combination of *L. plantarum* 299v and ZnSO4 on regulating the expression of *Slc30a2* and *IL-1*β is not different from that of the combination of *B. lactis* Ca360 and ZnSO4, but its effect on regulating the expression of *Slc39a4, Slc39a5, Slc11a2, MTF-1, Slc30a1, TNF-*α and *IL-10* is not as significant as that of the combination of *B. lactis* Ca360 and ZnSO4. Notably, in the present study, co-administration of probiotics *B. lactis* Ca360 and ZnSO4 demonstrated supior efficacy in modulating key zinc transportes, compared to the combination of *L. plantarum* 299v and ZnSO4, as evidenced by its potent restoration of *Slc39a5* and *Slc30a1* expression and suppression of *Slc39a14* and *TNF-*α. Previous studies have shown that *L. plantarum* 299v can enhance the bioavailability of minerals such as calcium, iron, and zinc (39). However, in this study, although the combination of *L. plantarum* 299v and ZnSO4 can enhance the bioavailability of zinc by improving the expression of zinc metabolism related proteins in zinc deficient mice, the effect is far inferior to the combination of *B. lactis* Ca360. The distinct profile indicates that probiotic *B. lactis* Ca360 has a more robust, strain-specific influence on regulatory network controlling zinc homeostasis.

Zinc deficiency induced a pro-inflamamtory dysbiosis in the gut microbiota, characterized by an enrichment of *Desulfobacterota*-like (including *Desulfovibrio, Bilophila*, and *Helicobacter*), and a depletion of homeostasis microbes—an observation consistent with prior studies (40). Combined intervention with *B. lactis* Ca360 effectively reversed these dysbiotic changes, and LEfSe analysis identified *Muribaculum* as a signature taxon in the *B. lactis* Ca360-treated group. Given its role in carbohydrate metabolism and SCFAs production, the enrichment of *Muribaculum* likely underpinned the zinc metabolism and the attenuation of inflammatory observed in this study (41). Notably, rather than restoring the microbiota to the NC baseline, B. lactis Ca360 induced a distinct compositional shift, with enrichment of Muribaculum and Bacteroidota.

**Figure 6:**
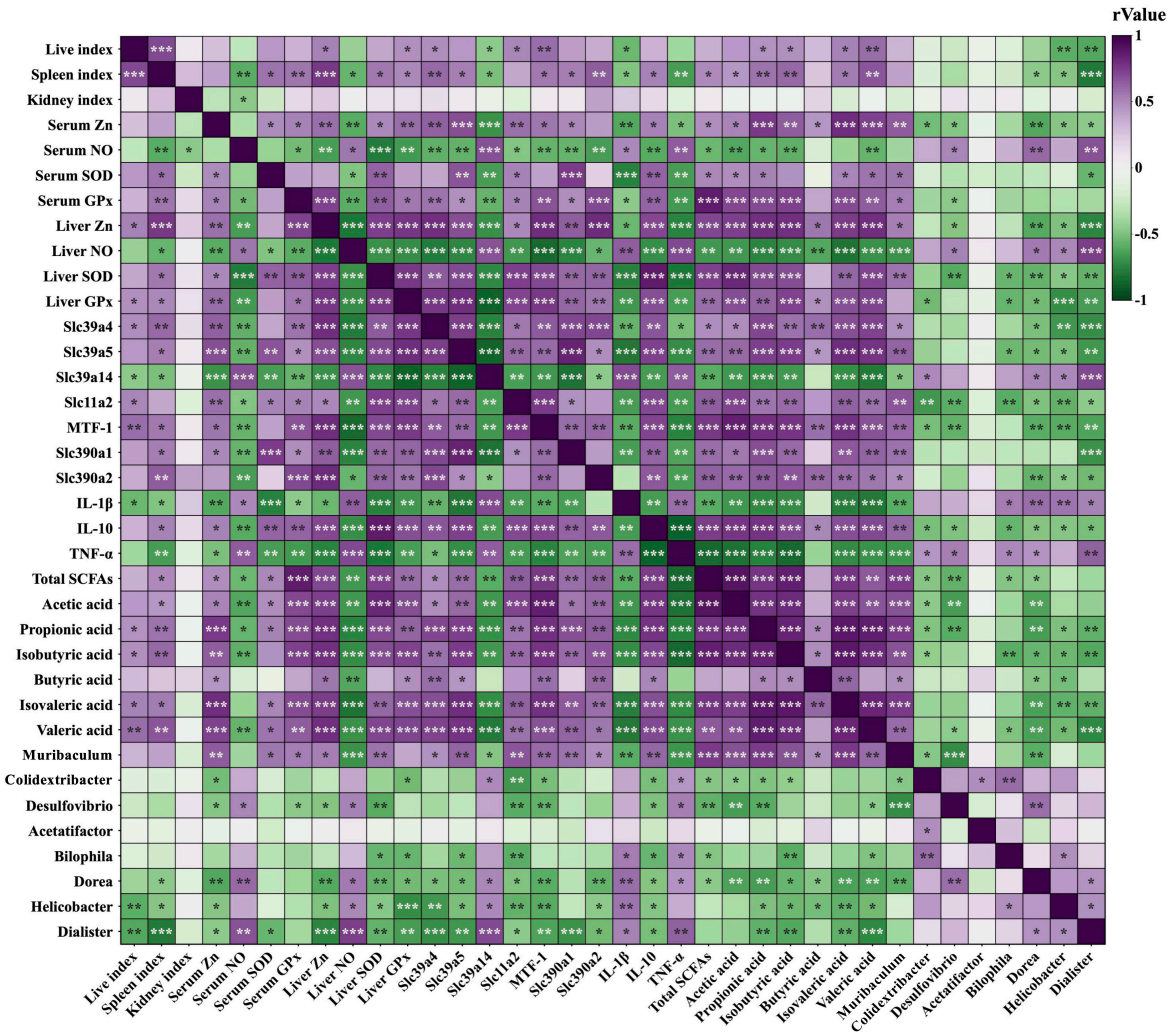
Spearman correlation analyses. Each group consisted of *n* = 10 mice. Significance levels are indicated as follows: *p < 0.05, **p < 0.01, ***p < 0.001.

Gas chromatography analysis results showed that compared with the zinc-deficient model group, the combined intervention of *B. lactis* Ca360 and ZnSO4 significantly increased the concentrations of total SCFA, acetate, propionate, isobutyrate, valerate and isovalerate in feces. This finding is highly consistent with the gut microbiota remodeling we observed. The intervention with *B. lactis* Ca360 significantly enriched the genus *Muribaculum*, which plays a key role in carbohydrate metabolism and SCFA production. Therefore, the increase in SCFA levels is likely a direct metabolite of *B. lactis* Ca360, regulating the composition of the gut microbiota and enriching beneficial functional bacteria (such as *Muribaculum*). The significant increase in SCFA concentrations, particularly acetate and propionate, in the *B. lactis* Ca360+ZnSO4 group can be directly attributed to the probiotic-driven enrichment of *Muribaculum*, a genus renowned for its carbohydrate-fermenting and SCFA-producing capabilities (as revealed by LEfSe analysis, Figures 4c-d). This metabolic shift, in turn, underpins the observed amelioration of colonic inflammation and improvement in zinc homeostasis, as SCFAs are well-established mediators of intestinal barrier integrity and anti-inflammatory responses. These SCFAs, especially acetate and propionate, are important signaling molecules for maintaining intestinal homeostasis (42). They not only provide energy for colonic epithelial cells but also enhance the expression of tight junction proteins, thereby reinforcing the physical barrier function of the intestine (43). In addition, SCFAs have significant anti-inflammatory and immunoregulatory effects (44). They can inhibit the production of pro-inflammatory cytokines (such as IL-1β, TNF-α) by suppressing histone deacetylase (HDAC) or activating G protein-coupled receptors (such as GPR41/43) (45). This is consistent with the molecular-level findings of this study: *B. lactis* Ca360 intervention can upregulate the expression of the anti-inflammatory cytokine IL-10 and downregulate pro-inflammatory genes such as TNF-α. Meanwhile, correlation analysis (Figure 6) also showed that the abundance of *Muribaculum* was positively correlated with good zinc status and antioxidant enzyme activity, but negatively correlated with pro-inflammatory indicators. Therefore, *B. lactis* Ca360 may jointly create a low-inflammatory and high-integrity intestinal microenvironment by remodeling the microbiota and increasing SCFA production. This improved gut environment not only directly alleviates local inflammation (such as colitis) but also indirectly promotes systemic zinc metabolism through the “gut-zinc axis.” A healthy intestine with fully functional barriers is more conducive to zinc absorption and the normal expression of transport proteins (such as slc39a4 and slc30a1), which partly explains why the combined intervention with *B. lactis* Ca360 is advantageous in restoring serum and liver zinc levels and enhancing antioxidant enzyme activities (SOD, GPx). In summary, the synergistic effect of *B. lactis* Ca360 and ZnSO4 on SCFA production is the key metabolic bridge linking their comprehensive benefits of “remodeling the gut microbiota” and “improving colonic barrier function, reducing inflammation, and ultimately correcting zinc-deficiency metabolic disorders.” This provides new experimental evidence for the development of *B. lactis* Ca360 as a probiotic strategy that manages zinc-deficiency-related diseases by targeted regulation of the gut microbiome.

To intergrate systemic responses, Spearman's correlation analysis was performed and it revealed a systematic link between gut microbiome and host zinc homeostasis the beneficial taxon *Muribaculum* was robustly correlated with improved zinc status (serum and hepatic Zn), antioxidant enzyme activity (SOD and GPx), and the expression of multiple zinc uptake and storage-related genes, and negatively with pro-inflammatory cytokines (*IL-1*β and *TNF-*α). This is consistent with previous research findings (46). Conversely, several genera (*Colidextribacter, Desulfovibrio, Bilophila, Dorea, Helicobacter*, and *Dialister*) showed significant positive correlations with NO and pro-inflammatory parameters, while exhibiting significant negative correlations with Zn content, antioxidant enzyme activity, and zinc transport-related gene expression. These results are consistent with research reports that increased *Desulfovibrio, Bilophila, Dorea, Helicobacter*, and *Dialister* can induce inflammation. Previous studies have shown that *Colidextribacter* has been identified as an inflammation associated gut microbiota and highly enriched in colitis mouse models (47). *Desulfovibrio*, a Gram-negative sulfate-reducing bacterium, may contribute to mucosal inflammation through hydrogen sulfide production (48). The excessive growth of *Bilophila* leads to dysbiosis and intestinal inflammation in mice, characterized by an imbalance in the gut microbiota and increased production of interleukin-10(49). *Dorea* has been found to increase intestinal permeability, which may induce translocation of microorganisms and microbial metabolites, thereby exacerbating liver inflammation (50). *Helicobacter* is closely associated with inflammation (51). The increase in the level of *Dialister*, a genus associated with inflammation, suggests a pro-inflammatory environment, which may accelerate tissue damage and carcinogenesis. These findings suggest that probiotic *B. lactis* Ca360 intervention may improve zinc homeostasis by remodeling the gut microbiota: suppressing pro-inflammatory, dysbiotic taxa while enriching beneficaial ones like *Muribaculum* and reducing the abundance of inflammation-related bacterial genera (*Colidextribacter, Desulfovibrio, Bilophila, Dorea, Helicobacter*, and *Dialister*), thereby creating a gut microenvironment more conducive to zinc absorption and homeostasis. Collectively, our findings advance the current understanding of probiotics in mineral nutrition and support the potential of *B. lactis* Ca360–based microbiome strategies for managing of zinc deficiency–related disorders. During the process of synergistic zinc intervention, the better effect of *B. lactis* Ca360 in zinc supplementation may be related to its ability to increase short-chain fatty acid concentrations, lower intestinal pH to enhance the solubility and ionization of minerals, thereby freeing minerals from food complexes and making them easier for intestinal cells to absorb, or its phytase concentration breaking down phytic acid and ‘releasing' bound minerals, making them absorbable and utilizable. This suggests that the probiotic's benefit may stem from steering the microbiota toward a novel, functionally beneficial configuration rather than restoring a pre-deficiency baseline.

Nevertheless, several limitations must be acknowledged to guide future translation. First, findings from the zinc-deficient mouse model require further validation in other animal models and, ultimately, in human clinical trials to confirm efficacy and safety. Second, the observed microbiota-host correlations, along with currently employed detection methods such as oxidative stress marker kits, necessitate more comprehensive and causal investigation through mechanistic studies—for example, supplementing with ELISA, Western blot, or other complementary assays. Third, while 16S rRNA sequencing and SCFA profiling offered initial insights, the functional role of key bacterial genera such as *Muribaculum* remains to be elucidated through more advanced techniques like metagenomics, metabolomics, or gnotobiotic approaches. Last, this study lacks a “probiotics-only” control group, making it difficult to distinguish the specific contributions of probiotics to zinc absorption promotion vs. direct anti-inflammatory/antioxidant effects. These shortcomings warrant refinement in subsequent research through both clinical investigations and mechanistic experiments.

Future studies could include fecal microbiota transplantation (FMT) in *B. lactis* Ca360–treated mice, non-intervention controls, or zinc-deficient mice to demonstrate causal roles of the modulated microbiota. Ultimately, efforts should focus on human trials to validate efficacy, and on mechanistic work to clarify causal microbiota–host interactions—paving the way for translating these insights into practical microbiome-based interventions for zinc deficiency, supported by real-world or clinical data on probiotic use and zinc status.

## 5 Conclusion

This study systematically evaluated the combined effects of *B. lactis* Ca360 and ZnSO4 in a murine model of zinc deficiency, demonstrating its efficacy in ameliorating zinc metabolism disorders, oxidative stress, and gut microbiota dysbiosis. By constructing a multi-level evidence chain and comparing it with *L. plantarum* 299v, we established a robust experimental foundation for its translational development of *B. lactis* Ca360. These findings underscore the dual potential of probiotic *B. lactis* Ca360 for clinical and nutraceutical applications.

## Data Availability

The data presented in the study are deposited in the NCBI Sequence Read Archive (SRA), accession number PRJNA1399062.
