# Supplementary material for: Synergistic effects of Bifidobacterium animalis subsp. lactis Ca360 and zinc sulfate on zinc transport, oxidative stress, and intestinal inflammation in zinc-deficient mice
Source: Front Nutr. 2026 May 28;13:1786466. doi: 10.3389/fnut.2026.1786466 (PMC13253517; doi:10.3389/fnut.2026.1786466)

Alpha and beta diversity

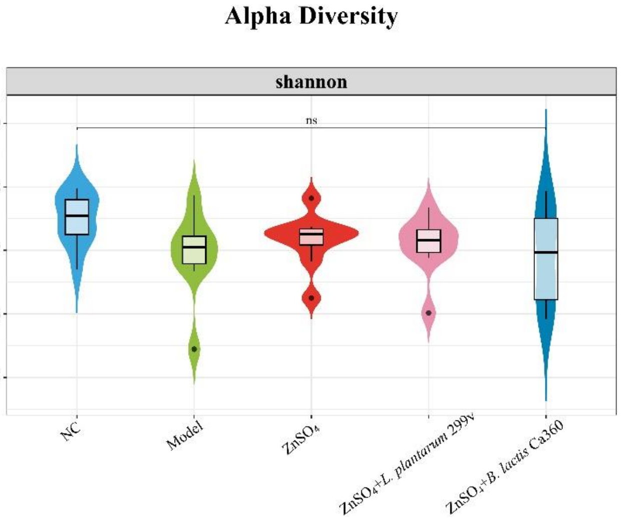

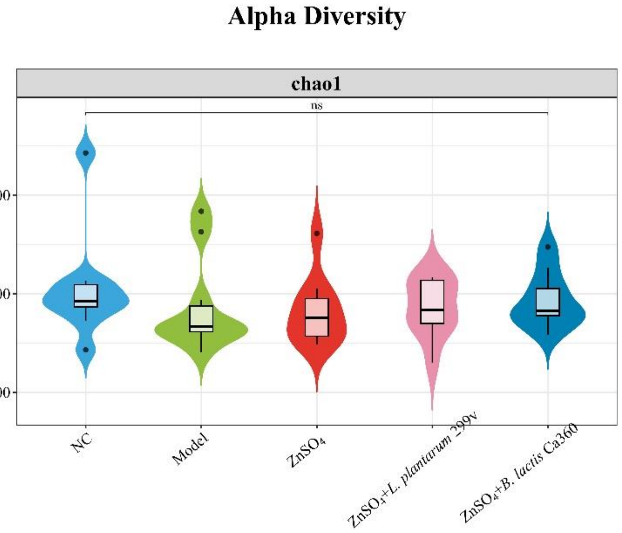

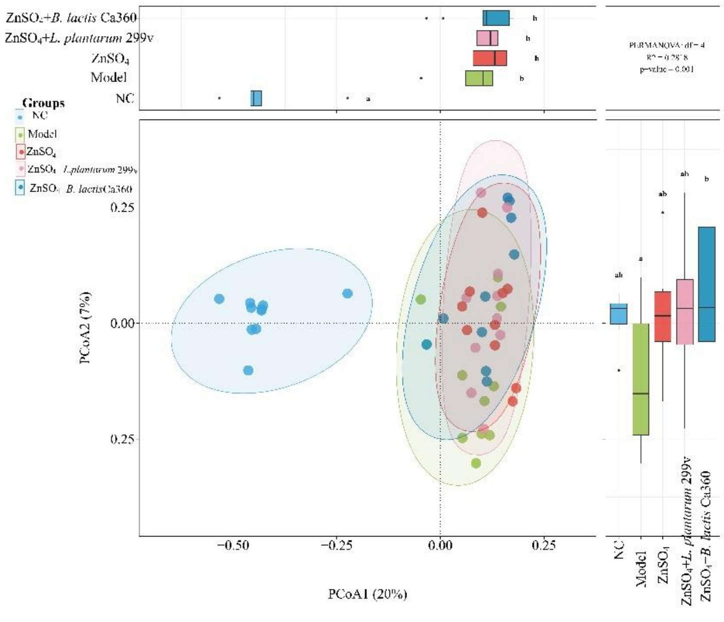

H&E staining of colon tissues from different groups

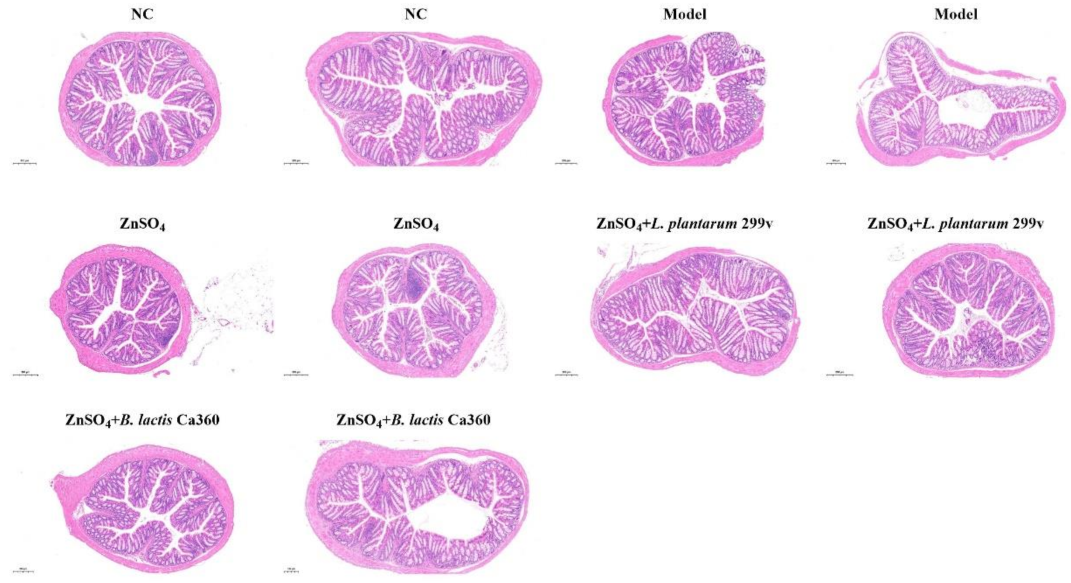

Supplement: Supplementary file 1 [file Data_Sheet_1.pdf]
